# Physiotherapy for Trismus Using Rocabado Exercises Following Mandibulectomy for Squamous Cell Carcinoma of the Lower Lip: A Case Report

**DOI:** 10.7759/cureus.31293

**Published:** 2022-11-09

**Authors:** Utkarsha R Mangulkar, Shubhangi Patil, Simran Y Jaiswal

**Affiliations:** 1 Community Health Physiotherapy, Ravi Nair Physiotherapy College, Datta Meghe Institute of Medical Sciences, Wardha, IND

**Keywords:** mouth opening exercises, rocabado exercises, lower lip, squamous cell carcinoma, physical therapy

## Abstract

Squamous cell carcinoma (SCC) is the most common malignant tumor of the oral cavity. Since it spreads and metastasizes more quickly than any other form of glabrous skin carcinoma, SCC of the lips seems to be more harmful. This report highlights the case of a 67-year-old man who underwent a mandibulectomy for lip SCC, subsequently having complaints of severe trismus and jaw pain, dysarthria, and difficulty drinking water. The patient's symptoms and trismus improved significantly via a course of physiotherapy. Six weeks of physiotherapy with various interventions such as mouth opening and closing exercises along with tongue protrusion, lower limb and upper limb passive movements, breathing exercises, lower limb mobility exercises, speech therapy, static shoulder exercises, static gluteus exercises, static hamstring exercise, and static quadriceps exercises, shoulder shrugs, neck isometrics, and shoulder-scapular sets, including goldfish exercises and Rocabado exercises may be helpful for managing symptoms such as trismus and other associative problems such as maintaining circulation and avoiding compensatory posture, pulmonary complications, and secondary complications, which may be helpful for managing the patient after mandibulectomy.

## Introduction

Oral cancer is the sixth most frequent cancer in the world. It is responsible for one-fourth of male and one-tenth of female malignancies in India [1]. Due to widespread tobacco chewing and smoking practices, oral cancer is very prevalent in India [2]. Additionally, oral cancer is more prevalent among people with lower levels of education, those who live in rural regions, those with poor dietary habits, and those who maintain poor oral-dental hygiene [3]. It is well-known that patients with head and neck squamous cell carcinoma (SCC) have a five-year survival rate of 40-60%. The probability of a tumour recurring is also significant, maybe as a result of maximum diagnosis in advanced stages (III and IV) [4]. Locoregional recurrence and distant metastatic disease have frequently been seen in 20-30% of individuals [5].

Lip carcinoma, one of the most curable types of head and neck cancer, is one of the most common forms of the disease. Lip exposure to sunlight is a substantial risk factor for cancer, unlike other mucosal surfaces of the head and neck [6]. SCC of the lower lip, a malignant tumor, constitutes 25-30% of all oral cancers [7]. When left untreated, lesions of the lower lip typically involve 40% of the lip and profoundly penetrate the muscle [8].

Surgery is recommended, mainly local excision of the lesion by mandibulectomy. Marginal and segmental mandibulectomy are the two forms of resection that may be distinguished for the mandible. Earlier, oral SCC that was close to or had invaded the mandible was treated by segmental resection [9]. Segmental mandibulectomy loses basal or lateral mandibular bone continuity, which has an adverse effect on phonation, mastication, and facial esthetics. Marginal mandibulectomy preserves this continuity [10].

The most frequent oral problems following surgery are trouble eating and impaired speech (dysarthria), and patients with head and neck cancer frequently experience restricted mouth opening, also known as trismus [11]. The most frequent postoperative oral crises include restricted mouth mobility, chest discomfort and dyspnea, edema, and limitations on activity around the surgical area. Physiotherapy may benefit those with oral cancer who are receiving treatment alternatives including head and neck exercises, mouth-opening exercises by utilizing therapeutic equipment, and a mouth proprioceptive neuromuscular facilitation approach. The main goal of a physiotherapy treatment program for postoperative patients is to help them regain functional range of motion (ROM) and so enhance their quality of life. The therapeutic method is to place patients in educational settings. Exercises for neck mobility, breathing techniques, mouth-opening exercises, and exercises including assistive devices, mouth proprioceptive neuromuscular facilitation (PNF), and bilateral upper-limb and lower-limb mobility exercises are just a few examples. Dr. Mariano Rocabado has examined temporomandibular dysfunction in great detail and created a 6 x 6 workout regimen, including six exercises performed six times per day [12].

## Case presentation

Patient information

**Figure 1:**
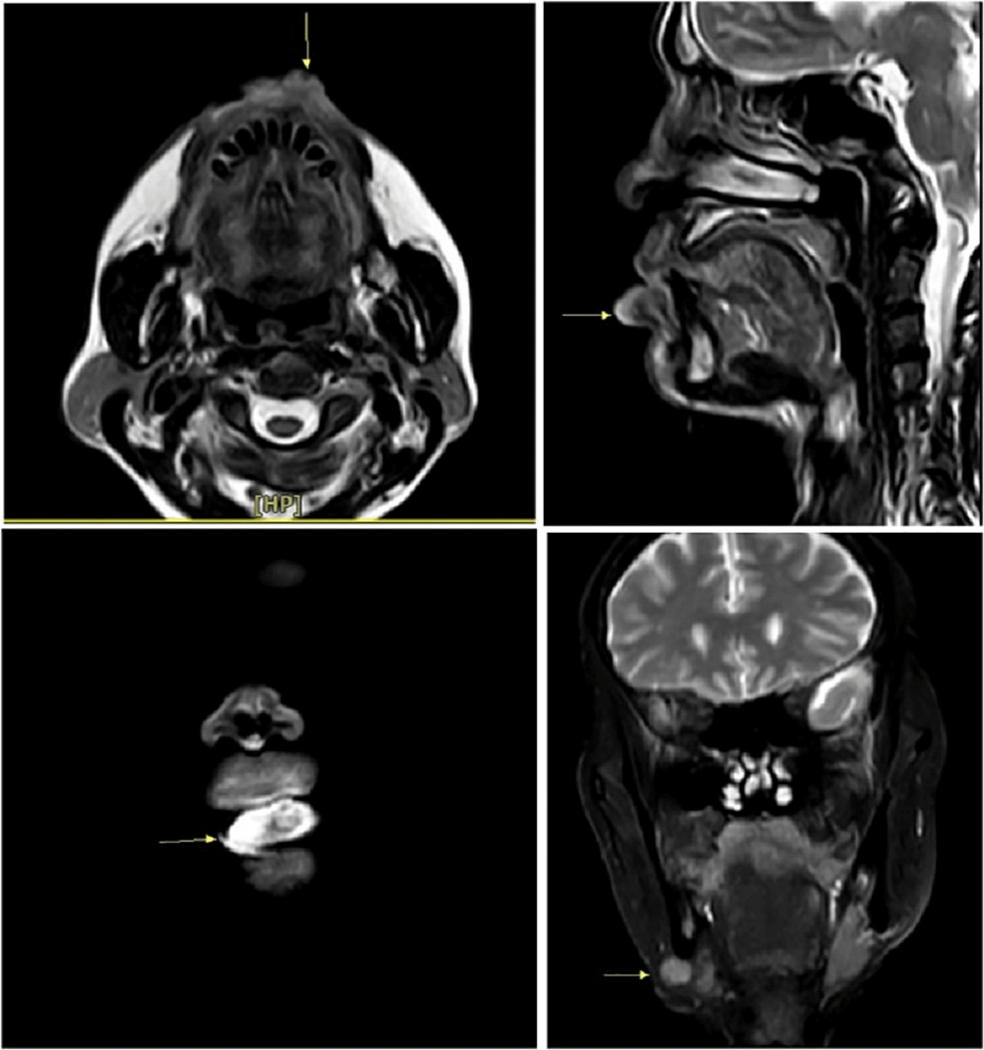
CECT of squamous cell carcinoma of the lip; the arrow indicates the site of the lesion top left: axial; top right: sagittal; bottom: coronal images CECT: contrast-enhanced computed tomography

A 67-year-old male had an ulcer over the right side of the lip, which was not healing for four months. The patient reported that his ulcer was initially small in size and gradually increased to its present size of 4x3 cm approximately. As reported by the patient, the pain was gradual in onset, intermittent, dull aching, and localized in nature. He also had a history of hypertension for seven years approximately and was on medication for the same. He also reported a history of tobacco chewing for 10 years. The patient presented to the hospital with these concerns and recommended tests, including magnetic resonance imaging (MRI), contrast-enhanced computed tomography (CECT) (Figure 1), and a biopsy, were completed. The patient was diagnosed as having well-differentiated lower lip SCC (lower right labiogingival sulcus). He was admitted to the hospital on August 2022 for surgical intervention where a segmental mandibulectomy was done and, a week later, SCC of the lower lip was surgically removed.

After surgery, the patient complained of difficulties with opening his mouth, was unable to speak, and was unable to do daily tasks like eating and drinking water. The pain was a dull aching in nature with an intensity of 8/10 on the Numerical Pain Rating Scale (NPRS). Jaw pain aggravated on mouth opening or in process of mastication, which was alleviated with avoidance of these activities. The patient was sent to the physiotherapy department for additional treatment due to all of these problems. The patient's concern was difficulty in post-operative mouth opening and inability to talk and inability to do activities of daily living.

Clinical findings

The patient was examined while sitting. Upon examination after the surgery, the patient's spine was upright and his hip and knee were 90 degrees flexed, and lying on the sofa. There was a nasal tube. A sensory examination was conducted as part of a neurological evaluation, and no loss of the superficial sense system (temperature, pinprick, and light touch) was discovered. On examination of vitals, pulse rate was 80 beats per minute, respiratory rate was 20 breaths per minute, and blood pressure was 140/90 mmHg. The pre-physiotherapy ROM of the temporomandibular joint (TMJ) or mouth opening was one finger.

Intervention

Post-Operative Goals

The short-term goals were to lessen discomfort, improve mouth mobility, improve mouth opening, and enable the patient to carry out daily activities. The long-term objectives were to maintain the mouth opening and mobility that had been attained. On the assessment of the patient by the physiotherapist, findings included restricted mouth opening, inability to communicate, and inability to do basic daily activities such as eating and drinking water.

Week 1

Mouth opening and closing exercises were taught along with tongue protrusion. Basic lower limb and upper limb passive movements were taught to improve and maintain circulation. Breathing exercises including thoracic expansion and deep breathing were taught to avoid pulmonary complications. Lower limb mobility exercises, including ankle-toe movements and heel slides to avoid secondary complications such as bed sores and deep vein thrombosis (DVT), were continued for one week.

Week 2

To improve the ROM of TMJ, exercises such as 6x6 (six exercises, six times) Rocabado exercise were taught, which includes functional jaw opening, controlled-ROM lateral deviation, lateral deviation and functional opening, protrusion ROM, self-stretch into opening, and self-distraction mobilizations. Goldfish exercises were also taught. In addition to this, a speech therapy referral was also given to recover speech difficulties in the patient.

 *Weeks 4-6*

Static shoulder exercises, static gluteus exercises, static hamstring exercises, and static quadriceps exercises are provided as isometric exercises for strengthening the upper and lower limbs. To prevent compensatory posture, shoulder shrugs, neck isometrics, and shoulder-scapular sets were taught. Exercises were taught to open and close the mouth while holding the opening open for 10 seconds using two ice cream sticks. Additionally, a mouth gauge gadget was utilized to help the patient expand his mouth wider. To prevent respiratory difficulties in the patient, breathing techniques such as deep breathing, deep diaphragmatic breathing, and pursed lip breathing were taught. We kept doing the goldfish and Rocabado 6x6 routines. To prevent errors in jaw opening, all activities involving opening the mouth were performed in front of a mirror.

Home program

**Figure 2:**
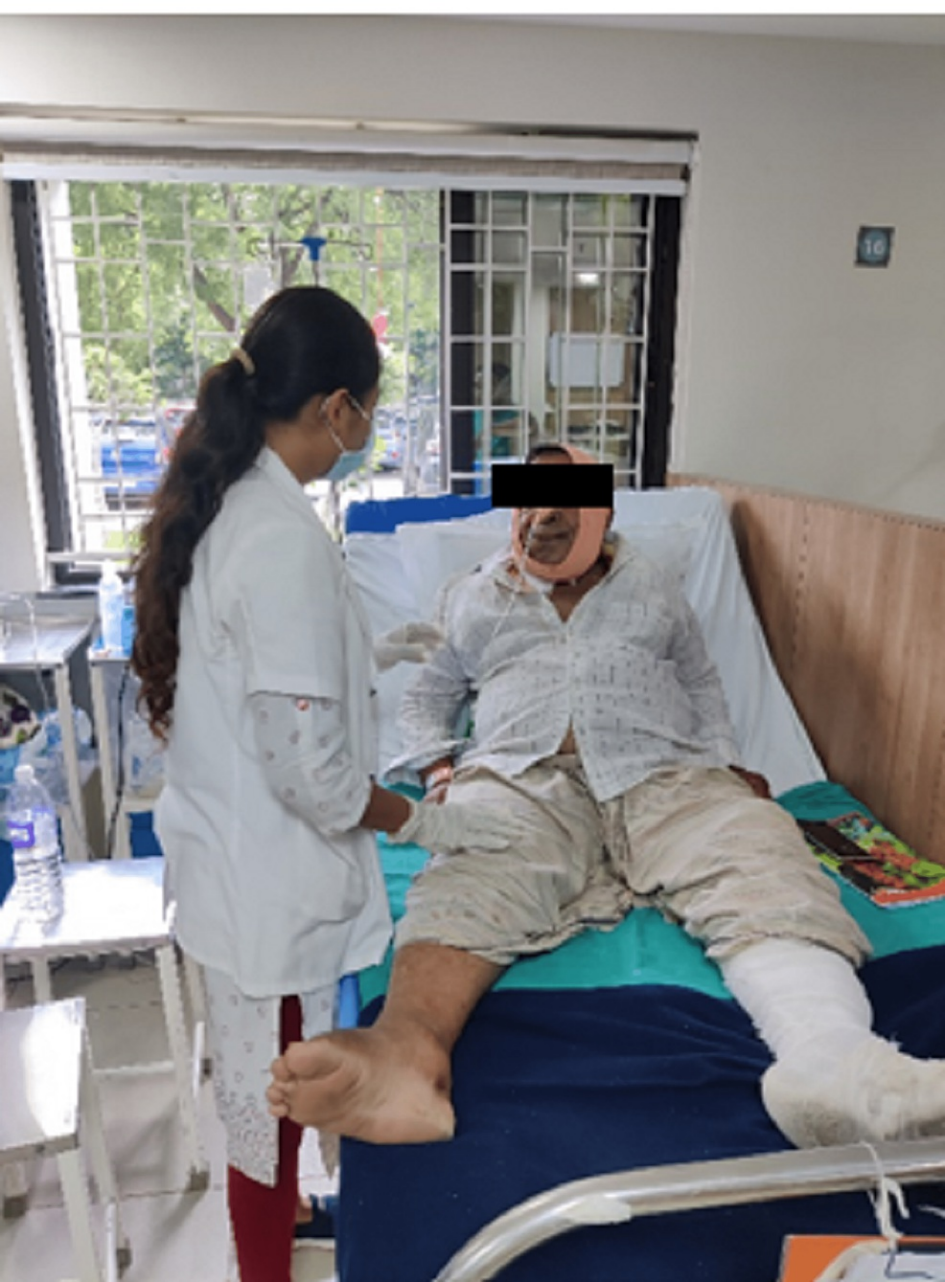
Explaining the treatment to the patient

**Figure 3:**
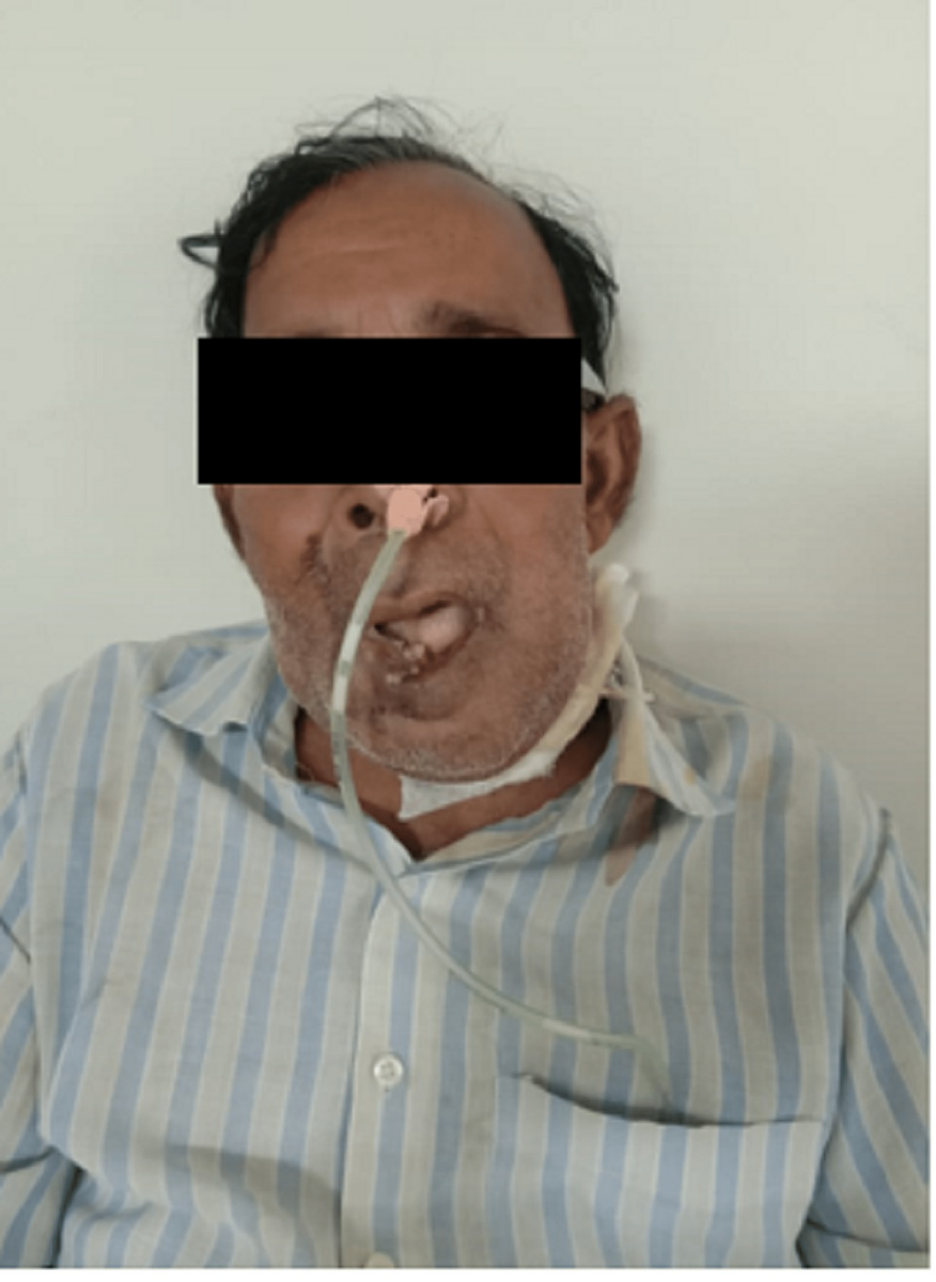
Mouth opening improved after treatment

Home exercises were taught to the patient to perform after the activities after the discharge, with the continuation of all the exercises taught to the patient, with proper repetitions. Mouth opening was seen to be increased and there was a reduction in pain. The treatment was explained to the patient (Figure 2). Mouth opening increased after treatment (Figure 3).

Follow-up and outcome

The patient experienced no discomfort or difficulties and was able to complete all activities of daily living. Additionally, post-physiotherapy TMJ (mouth opening) was increased to three fingers. The patient was motivated and eager to participate in the physical treatment. The patient has also received a home program briefing and advice to modify their posture. Post-operative day 2 received an 8/10 NPRS rating. Improvements in NPRS were seen to be 2/10 after six weeks of physiotherapy intervention post surgery. An increase in the patient's shoulder and mouth opening ranges indicated improvements. As the patient continued with physiotherapy treatment at home, improvements were progressively seen.

## Discussion

This case report describes a 67-year-old patient’s complete rehabilitation protocol after undergoing surgery of SCC of lower lip left side with segmental mandibulectomy from 31 to 37, bilateral modified radical neck dissection, and reconstruction with free fibula flap of the left side and right side Estlander flap under general anesthesia.

Restricted mouth opening is the leading post-surgical impairment seen in patients with carcinoma of the lip that causes difficulty in activities of daily living [11]. Physiotherapy plays a crucial role in treating patients with restricted mouth opening through various interventions such as Rocabado exercises and goldfish exercises. This improves the patients' condition and makes them independent to do basic activities of daily living [12]. Preventive and therapeutic measures are taken to improve trismus in patients. The preventive measures include TheraBite® (Atos Medical AB, Malmö, Sweden), active ROM exercises (with or without chewing gum), and wooden tongue depressors. Dynamic bite openers, a homemade sledgehammer device, TheraBite, active range of motion exercises, the Engstrom jaw mobilizing device, tongue depressors, rubber plugs, dental treatment stimulations, and many different stretching techniques were seen to be therapeutic measures. There are many different stretching techniques that were seen to be therapeutic measures. Mouth-opening variations were very diverse. However, no exercise method can be said to be obviously better than others for either preventing or treating the trismus that results from head and neck cancer. After exercise therapy, both therapeutically and preventively, mouth opening increased. Early therapy initiation and exercise compliance appear to be crucial for successful outcomes [13]. It was analyzed retrospectively that the effects of exercise therapy on trismus related to head and neck cancer or as a consequence of its treatment indicate that mouth opening increases significantly after exercise therapy in patients with trismus [14].

## Conclusions

This case highlights an older man who underwent a mandibulectomy for lip SCC and subsequently had severe trismus and an inability to talk or drink water. The patient improved significantly and regained jaw-opening mobility following a course of physiotherapy featuring Rocabado exercises. These exercises should be considered for patients with head and neck symptoms such as trismus following mandibulectomy.
